# Individual migration timing of common nightingales is tuned with vegetation and prey phenology at breeding sites

**DOI:** 10.1186/1472-6785-14-9

**Published:** 2014-03-21

**Authors:** Tamara Emmenegger, Steffen Hahn, Silke Bauer

**Affiliations:** 1 Department of Bird Migration, Swiss Ornithological Institute, Seerose 1, Sempach 6204, Switzerland; 2 Conservation Biology Division, Institute of Ecology & Evolution, University of Bern, Baltzerstrasse 6, Bern 3012, Switzerland; 3 Department of Animal Ecology, Netherlands Institute of Ecology (NIOO-KNAW), PO Box 50, Wageningen NL-6700 AB, The Netherlands

## Abstract

**Background:**

The timing of migration substantially influences individual fitness. To match peak requirements with peak resource availability, we hypothesized that individual migrants schedule spring migration in close relation to seasonal changes in environmental conditions along the route and particularly, at the breeding destination.

To test this hypothesis, we investigated the timing of spring migration in male common nightingales *Luscinia megarhynchos*, a small Palearctic-African long-distance migrant, by linking spring migration timing to the phenology of local environmental conditions at non-breeding migratory stopover and breeding sites. In particular, we related individual migration decisions (i.e. departure and arrival) of nine males to site-specific vegetation phenology (based on remotely sensed vegetation index) and a proxy of food availability (based on insects’ thermal requirements).

**Results:**

We found weak relation of departures from non-breeding and no relation of stopover timing with local phenology. However, our results showed that individuals, which departed early from their non-breeding sites and arrived early at the breeding site closely matched spring green-up there. Early arrival at the breeding site meant also a close match with peak food availability for adults and in a time-lagged manner, for offspring.

**Conclusion:**

Our findings suggest that male nightingale used cues other than local phenology for their departure decisions from non-breeding grounds and that there is some evidence for equalizing late departures during the course of migration.

## Background

Annual migration between non-breeding and breeding sites is a widespread phenomenon in various animal taxa [1]. As almost all places on earth are seasonal to some degree, the timing of migration and other subsequent life-history activities has strong fitness consequences [2,3]. This applies particularly to the migration from the non-breeding to the breeding grounds (hereafter referred to as spring migration), for which arrival time and arrival body-condition on the breeding grounds are likely correlates of reproductive success and survival [4]. For instance, late arrival at the breeding site may come at the cost of the best territories already being occupied [5] or the individuals might release offspring not fully prepared to master the challenges of migration to non-breeding areas [6]. In contrast, too early arrival may expose migrants to adverse weather conditions and risk of starvation [7]. Similarly, individuals arriving in good body condition may cope better with adverse conditions and invest more in offspring [8].

However, arrival time and body condition in the breeding grounds are mainly determined by conditions encountered during migration, at intermittent stopover sites or even at non-breeding grounds [9,10]. Ideally, migrants should time arrival on any stationary site with favourable conditions and, particularly in the breeding grounds, match peak requirements with peak resource availability [11,12]. This, in turn, would require that migrant animals can predict the (future) development of conditions at sites over longer distances and possibly across ecological barriers. Although specific cues involved have remained largely elusive, there are indications that migratory birds are indeed able to tune migratory progression with the development of resources. For instance, passerine birds adjusted travel time according to conditions en route [13] and aerial-feeding insectivores can time arrival at the breeding site in accordance to the local vegetation phenology in the non-breeding grounds [14]. Furthermore, the annual variation in mean passage time in the Mediterranean area was positively correlated with primary production in both departure region and passage areas in several trans-Sahara migrants [10]. Similarly, favourable environmental conditions in non-breeding areas have resulted in late arrival at the breeding sites and good environmental conditions during stopover in early arrival at the breeding sites [15,16].

Thus, there is some evidence that birds used cues, which correlate with conditions at sites ahead or even the phenology of resources at their breeding sites from non-breeding areas. The latter was attributed to factors that influence large-scale climatic patterns, e.g. the North Atlantic Oscillation (NAO), which enabled long-distance migrant passerines predicting conditions at European breeding sites from their non-breeding grounds in Africa (e.g. [17]). However, while the migration phenology of some species advanced during the last 20 years, the migratory behaviour of other migrants relies on endogenous rhythms or is driven by environmental factors which are not affected by climate change [18,19].

However, most above-mentioned studies investigated migration timing at the population level [10,13] and/or considered only specific parts of the spring journey [20]. Notable exceptions are individual-based studies in migratory geese [21,22], which were shown to closely track the vegetation growth during spring migration (the so-called “green-wave hypothesis”, [23]). Thus, we know presently very little about how individuals time their entire spring migration, especially in animals other than herbivores and those that migrate across ecological barriers.

Therefore, we investigated the individual timing of spring migration in a small insectivorous Palearctic-African long-distance migrant, the common nightingale *Luscinia megarhynchos* (Brehm), in relation to the phenology of the local environmental conditions from its African non-breeding to its European breeding sites.

We hypothesised that migration timing is tuned with phenology along individual migration routes and tested this hypothesis with measures derived from the Normalized Difference Vegetation Index (NDVI) and temperature. In particular, arrival at and departure from sites of residency should coincide with particular local phenological events such as spring green-up or change in NDVI (at the specific site). Additionally, we tested whether the timing of spring migration has a consequence on projected subsequent reproductive timing. Therefore, we additionally linked the arrival at the breeding site with the local onset of insect availability as well as the offspring’s peak food requirements with the period of high food availability.

Here, we expected the males to arrive after the onset of food availability at the focal site. We considered reproductive timing to be optimal, if the period of insect larvae availability encompasses the period of peak energy requirement of offspring.

## Methods

### Migration routes and schedules

We investigated migration in individual nightingales originating from three European populations in Bulgaria (42.1°N, 27.9°E), in Italy (44.6°N, 11.8°E; south of the Alps), and in eastern France (47.6°N, 7.5°E; north of the Alps). An earlier study suggested that these populations are representative of birds using the western, central and eastern flyways for crossing the Mediterranean Sea and Sahara desert [24]. Birds were captured during the breeding season in 2009, equipped with leg-loop mounted geolocators (SOI-GDL1, Swiss Ornithological Institute; mean mass: 1.12 g including harness = 4.8% of average adult body mass) and recaptured in spring 2010, for details see [25] and Additional file 1: Table A1. This yielded complete spring migration data, i.e. from non-breeding site departure until arrival at breeding sites of nine individuals (males) from Bulgaria (B1-4), Italy (I1) and France (F1-4).

We derived timing and positions from recorded light intensities using the following standard procedure [26]: a) Definition of sun-events (light level threshold = 3 arbitrary light units); b) Identification of timing and stationary periods (minimal length of stationary periods = 3 days, change point probability threshold = 90% quantile), in particular departure from non-breeding sites, the arrival at and departure from the spring stopover sites as well as the arrival at the breeding sites and c) calculation of positions. Please note that although geolocation can by definition not determine spatial positions during equinox, the timing of migratory steps can still be identified [26,27]. For more detailed background information on data analysis see Additional file 1: Table A3 and Additional file 2.

For all individual stationary periods, we estimated the Kernel density from the point locations (ESRI ArcGIS 9.3, search radius: 200 km). We defined a “stationary site” as the polygon area within 90% Kernel density isopleth. When stopover sites were composed of more than 50% unsuitable habitat (e.g. bare or sparsely vegetated areas) according to reclassified data from the Global Land Cover 2000 database (European Commission, Joint Research Centre [http://bioval.jrc.ec.europa.eu/products/glc2000/glc2000.php]) we selected only suitable habitat within the 60% isopleth as stopover site.

### Vegetation phenology

**Figure 1:**
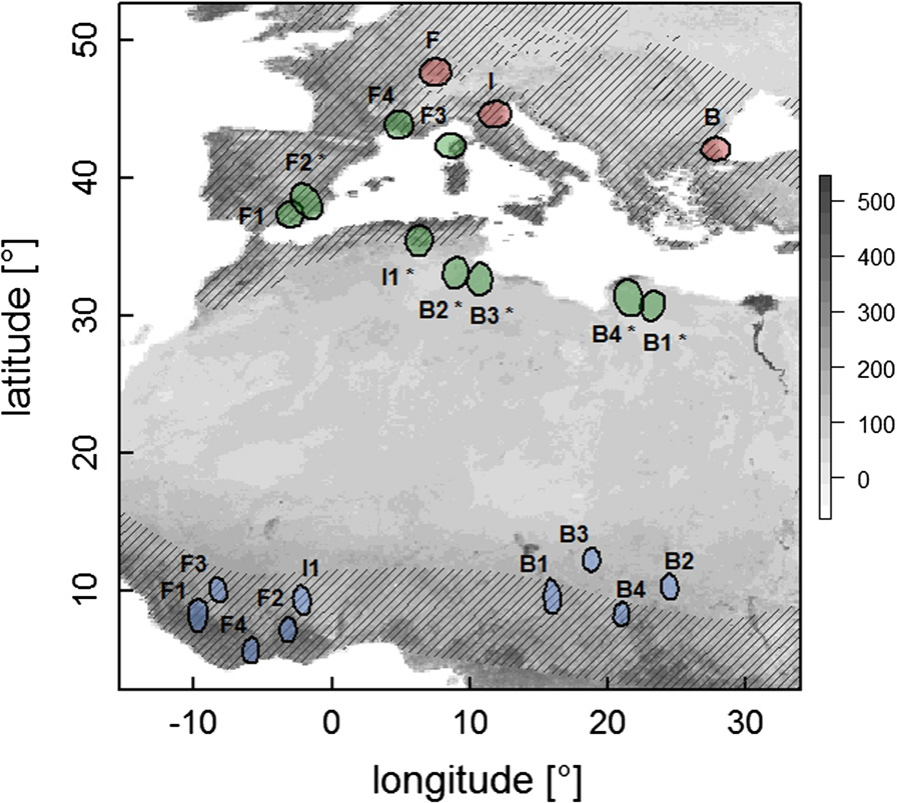
**The spring migration routes of nine common nightingales.** The polygons depict 90% kernel density isopleths of non-breeding (blue), stopover (green) and breeding sites (red) of the males from three populations (B = Bulgaria, I = Italy and F = France). Stopover sites composed of more than 50% unsuitable habitat are marked with asterisks. The currently estimated breeding and non-breeding distribution of the nightingale, according to [29], covers the shaded area. The grey-scale of the background indicates the NDVI (white = non-vegetated, black = dense green vegetation) of the first week of the year 2010.

We used data of the Normalized Difference Vegetation Index (NDVI) to characterize seasonal patterns in primary productivity at all stationary sites [28]. We processed NDVI data originating from the National Oceanic and Atmospheric Administration (NOAA [ftp://ftp.orbit.nesdis.noaa.gov/pub/corp/scsb/wguo/GVIx/GVIx_VH_16km/NVI]) – in particular, we selected the area 28°W to 57°E and 75°N to 55°S, applied a land-sea mask and aggregated the 16×16 km grid to a 64×64 km grid (for an example see the background of Figure 1). Afterwards, we extracted the weekly NDVI for the year 2010 and calculated the mean NDVI for each stationary site. To identify the relation between the migration timing and site-specific phenology we calculated the mean change in NDVI during the two weeks around arrival and departure on each particular stationary site.

In addition, we defined the date of spring green-up for all breeding sites by determining the week of maximum slope in NDVI phenology data in spring using a logistic regression [30]. We used the time difference between arrival date and spring green-up to measure how individuals hit the local vegetation phenology.

### Insect phenology at the breeding sites

Nightingales, as ground-dwelling insectivorous passerines, mainly rely on the availability of small-sized insects of the orders Coleoptera, Diptera, Hemiptera, Hymenoptera and Lepidoptera on the ground substrate and litter as well as on protein-rich insect larvae to feed their offspring [31]. As direct measures for the (local) phenology and abundance of insects and their larvae are usually not available but as insect development is temperature driven we estimated their appearance from local temperature data based on the compilation of thermal requirements of 835 species, provided by Jarošík and colleagues [32] see [https://secure.fera.defra.gov.uk/pratique/downloadItem.cfm?id=491]). To this end, we calculated two measures that indicate the time when hibernating insects and larvae become available and the period over which (newly produced) insect larvae might be available. The first can be estimated by determining the date at which the base development threshold of 10.4°C mean daily air temperature is reached.

For the second measure, we determined the time period when insect larvae become abundant from accumulated temperature (degree days), i.e. the ambient temperatures exceed the base development threshold over a specific time. The period of larvae availability starts when the accumulated temperature for hatching (28.5 degree days) is reached. It ends, when accumulated temperature for a complete larval development (328 degree days) is reached. We used daily mean temperatures from near surface air temperature data of 2010 provided by the National Centers for Environmental Prediction [33], derived for the three breeding locations using R package RNCEP [34].

Finally, we estimated the period, during which the offspring food requirements peak. Egg-laying starts approx. 6–12 days after arrival, 4–6 days are needed for clutch completion and 13 days for incubation [35-37]. Additionally, the daily energy requirement of the offspring reaches its highest value towards fledging approx. at day 7 for insectivorous passerines with a weight of about 21 g [36-38]. This resulted in the offspring’s energy requirements to peak between 30 and 38 days after their parents’ arrival at the breeding site.

We evaluated how arrival at the breeding grounds matched the development of food resources both for adults and offspring by (1) relating date of arrival to date when the insects’ base development threshold was reached and (2) calculating the time difference between the end of offspring’s peak food requirement and the end of larvae availability.

When assessing whole-part correlations, e.g. between individual date of arrival at the breeding sites and the time difference between the arrival at the breeding site and the local spring green-up, we avoided any artificial restriction to positive r-values following the argumentation of [39,40]. The analysis of geolocator was done in the R package GeoLight [41]. All analyses (t tests and ordinary linear regression) were performed in R 2.14.× [42].

## Results

### Migration routes and schedules

We identified the migration routes (Figure 1) and schedules (Additional file 1: Table A2) of in total nine individuals. The non-breeding sites of the nine individuals were located in sub-Sahelian Africa, stretching from African west coast to the eastern border of Sudan. All individuals used intermittent stopover sites in the Mediterranean region ranging from Spain, southern France and Corsica to northern Algeria, Tunisia and Libya. On average, birds left their non-breeding sites on 23 March (range 18–27 March), arrived at their Mediterranean stopover sites on average on 31 March (range 28 March-8 April), where they stayed for approx. 13 days (5–20 days), and arrived at the breeding sites on average on 19 April (range 14–26 April). Thus, spring migration lasted on average 27 days (20–33 days).

### Vegetation phenology

**Figure 2:**
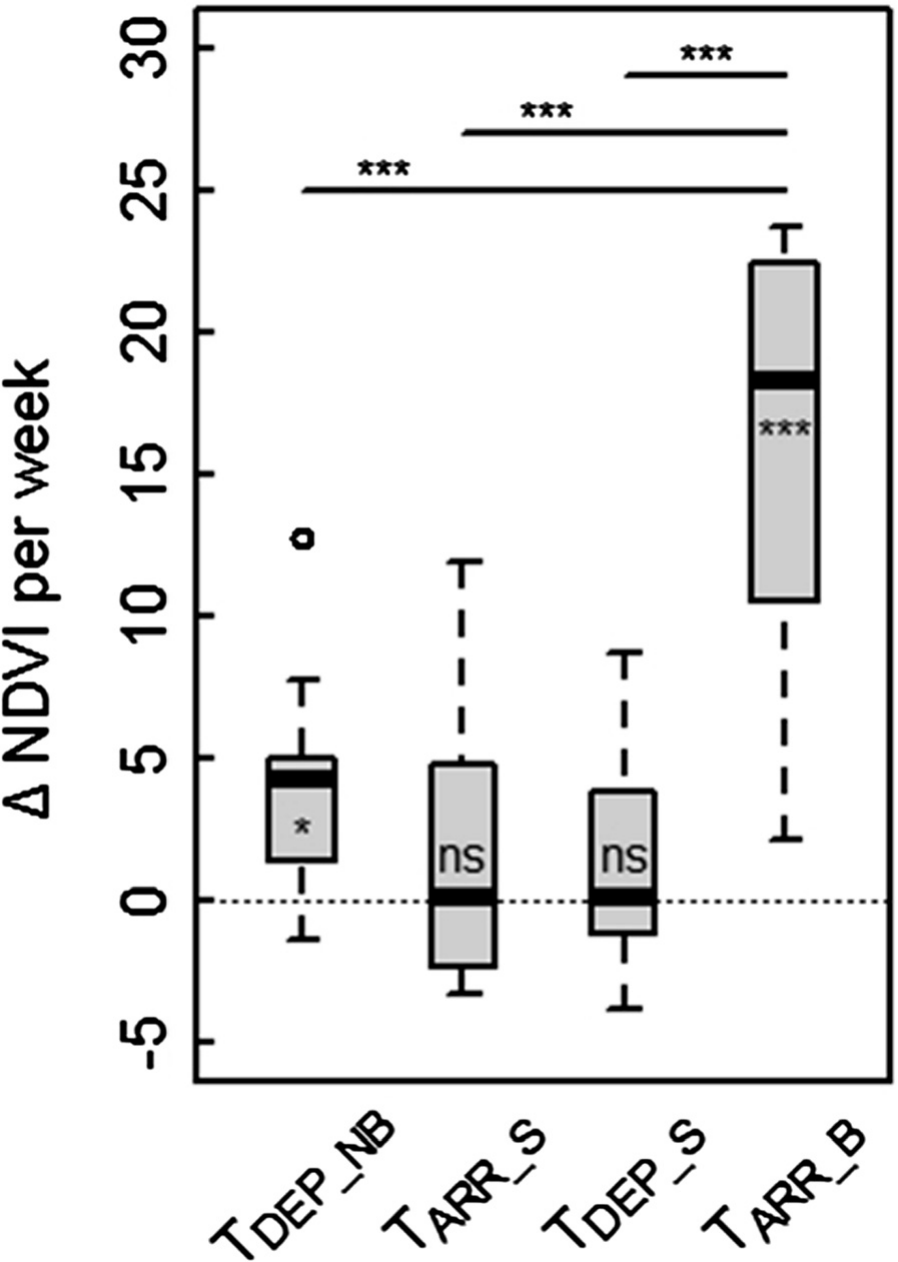
**Change in NDVI during two weeks around migration events of the nine male nightingales.** Departure from non-breeding site (T_DEP_NB_), arrival at (T_ARR_S_) and departure from the spring stopover site (T_DEP_S_) as well as arrival at breeding site (T_ARR_B_). Three asterisks and one asterisk indicate high (p ≤ 0.001) and moderate (p ≤ 0.01) significance of difference from zero in a *t*-test, respectively.

**Figure 3:**
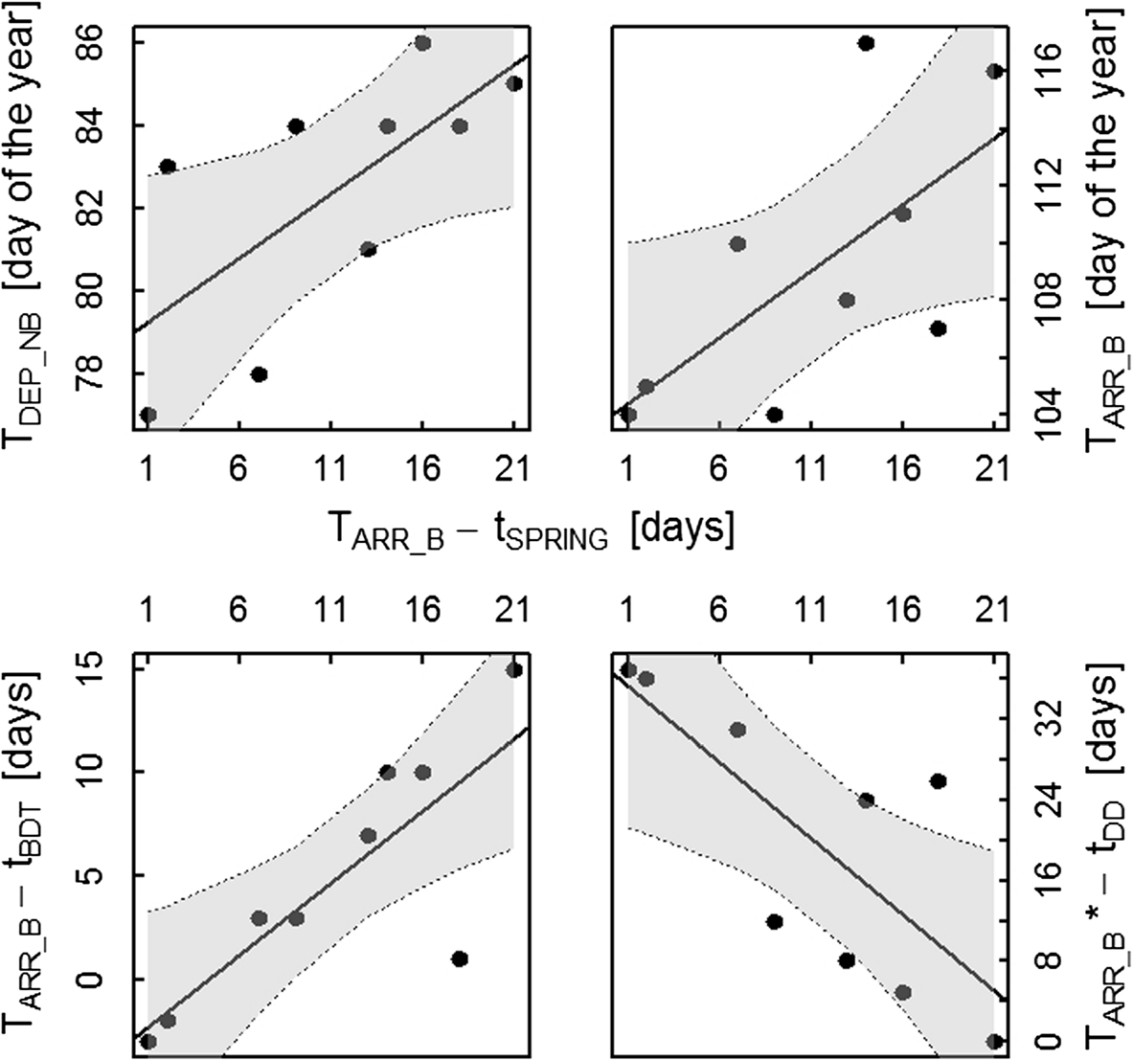
**Correlations between breeding site phenology and the timing of individual migration events.** The time difference between arrival and spring green up at the breeding site (T_ARR_B_ – t_SPRING_) in relation to migration timing (top left and right) and to resource phenology at the breeding site (bottom left and right). Significant positive relation between T_ARR_B_ – t_SPRING_ and departure from the non-breeding site (T_DEP_NB_; top left), arrival at the breeding site (T_ARR_B_; top right) and time difference between arrival and the date when insects start to develop at the breeding site (T_ARR_B_ – t_BDT_; bottom left). Significant negative relation between T_ARR_B_ – t_SPRING_ and the time difference between the end of the offspring’s peak food requirement and the end of larvae availability (T_ARR_B_* – t_DD_; bottom right). At each solid regression line the corresponding confidence interval (grey shaded area) and confidence limits (dotted lines) are given. For the test statistics see main text.

Mean changes in weekly NDVI during two weeks around the date of arrival at and departure from the spring stop-over site were not significantly different from zero (mean_ARR_S_ = 2.23 ± 5.27 SD, t = 1.27, df = 8, p = 0.24; mean_DEP_S_ = 1.63 ± 3.94 SD, t = 1.24, df = 8, p = 0.25; Figure 2). In contrast, the mean weekly NDVI changes during departure from the non-breeding sites (mean = 4.26 ± 4.17 SD, t = 3.07, df = 8, p = 0.02) and arrival at the breeding grounds (mean = 16.18 ± 7.77 SD, t = 6.24, df = 8, p < 0.001) were significantly larger than zero. Both departure from non-breeding sites (r^2^ = 0.47, df = 7, p = 0.04; Figure 3) and arrival at the breeding sites (r^2^ = 0.45, df = 7, p = 0.05; Figure 3) were positively related with time difference between the arrival at the breeding site and the local spring green-up such that individuals departing early from the non-breeding site and arriving early at the breeding site matched spring green-up closest.

### Insect phenology at the breeding sites

Arrival at the breeding grounds was closely related to the difference between arrival and the date when the base developmental threshold was reached, i.e. early arriving individuals also arrived shortly after insects became available (r^2^ = 0.74, df = 7, p < 0.001). In contrast, stop-over duration was only slightly related to the time difference between arrival and the date when the base developmental threshold was reached (r^2^ = 0.34, df = 7, p = 0.1). Thus, how closely individuals matched arrival with insect availability was independent of whether they stayed longer or shorter on stop-over sites. The period between the end of offspring’s peak food requirement and the end of larvae availability correlates with the departure from the non-breeding sites (r^2^ = 0.39, df = 7, p = 0.07), so that birds leaving their non-breeding sites early, profit from a longer period of high larvae availability after the period of the offspring’s highest food requirements. Additionally, the time difference between the arrival at the breeding site and the date when the base development threshold was reached was negatively related to the period between the end of offspring’s peak food requirement and the end of larvae availability, i.e. if individuals arrived close to the day when base development threshold was reached, the period of high larvae availability after the period of the offspring’s highest food requirements was long (r^2^ = 0.69, df = 7, p = 0.01). Finally, the individual time difference between arrival and spring green-up at the breeding site was related with the individual time difference between arrival and the date when the base development threshold was reached (r^2^ = 0.66, df = 7, p = 0.01; Figure 3) as well as the time between the end of offspring’s peak food requirement and the end of larvae availability (r^2^ = 0.57, df = 7, p = 0.02; Figure 3).

## Discussion

For the nine male nightingales in our study we found the timing of only one migration event namely arrival at the breeding site to be significantly linked to NDVI phenology there. In contrast, departure from non-breeding sites was only very weakly linked to local NDVI phenology. All other migration events such as arrival or departure from stopover sites were not related to any of the local phenology measures we tested. These results stem from only nine individuals and future studies with larger sample sizes are required to confirm our findings.

Our results are in contrast to the expectations and suggest that male nightingales used other cues for the departure decisions from the non-breeding sites. For the departure from the non-breeding sites, photoperiod might be the most important trigger. Indeed, photoperiod has been identified as a universal cue triggering (preparations for) migration in the majority of migratory species across all taxa [43], and even marginal changes of photoperiod can be sufficient to trigger preparation for migration in migrants wintering at low-latitudes [44]. The fact that departures from non-breeding sites in our study showed the lowest variability also suggests a prominent role of photoperiod for the onset of migration. This also points out to a potential threat: if climatic changes differ in magnitude and direction between the various places migratory birds use [45], birds relying on the invariant photoperiod might mistime various activities in their annual cycle. For instance, departing too late from the non-breeding areas can have cascading effects, with mistimed arrival in the breeding grounds, a large mismatch between peak food availability and peak food requirement of offspring, and thus, negative fitness consequences [46]. Hence, relying only on photoperiod for timing of migration might become an ecological trap [47] and has been discussed as one major factor for the declines of many species within the Palaearctic-African migration system [48]. It has sometimes been argued that migrants could catch up in the course of migration even if departure from the non-breeding site was non-optimal (usually: too late), e.g. by changing migration route or speed, but often migration strategy remained unaffected by climate trends [49].

Surprisingly, we also found no behavioural response to environmental conditions en route. Arrival at and departure from stopovers were unrelated to local phenology suggesting that birds used stop-over sites regardless of the progress of vegetation phenology at the focal site. This may be explained by a) differences in quality of sites and resulting differences in fuelling rates, b) carry-over effects of site-quality/fuelling rates in earlier sites, c) methodological issues inherent to geolocation and our assumed relation between NDVI and insect abundance (see below).

Accordingly, we infer, that fuel deposition rate diverged widely among the different individuals or stopover sites they visited. Fuel deposition rate depends on many factors, e.g. food abundance and accessibility, weather, predation risk as well as competition and individual state [50]. Furthermore, we found that arrival at the breeding sites was related to stopover duration, i.e. individuals that stayed shorter at stopover sites also arrived earlier at their breeding sites, regardless of total migration duration. Although it remains elusive at this stage why staging times at stopover sites differ between individual nightingales, there are several non-exclusive explanations: First, the quality of stopover sites in terms of re-fuelling could differ substantially and individuals in “high-quality” sites may rapidly replenish their body reserves in preparation for the next migratory step. Second, individual condition on arrival at a stopover site might be co-determined by the quality of preceding sites, which affects their requirements at the present site (carry-over effects, see [51]). If a spatial autocorrelation in quality between successive sites exists, migrants might adjust the timing of migratory progression according to conditions at sites ahead [52]. Unfortunately, our results are inconclusive here – we found no significant relation between absolute NDVI data and individual differences in four measures of timing (e.g. the date of arrival at the breeding site, the time difference between arrival at the breeding site and local spring green-up, the time difference between the arrival at the breeding site and the date when the insects’ base development threshold was reached, and the period between the end of offspring’s peak food requirement and the end of larvae availability) suggesting the latter not being explained by differences in productivity between stopover sites.

Moreover, we found that arrival at breeding sites was related to proxies of food availability for adults and their offspring. Birds arriving temporally close to the onset of insect availability also enjoyed a long time period of high larvae availability after the offspring’s peak food requirement, which implies that covering the food requirements of arriving males and offspring is not a mutually exclusive task. The timing of arrival of adult birds in the breeding grounds can importantly influence the fate of their offspring and thus, their reproductive success [49].

For all individuals of our study populations, the periods of high availability of larvae covered the periods of high energy requirements, suggesting no apparent mismatch between food supply and demand. Possibly, this is facilitated by wide foraging niche and a seasonally flexible foraging strategy in nightingales [25]. However, in dietary specialist species, like the pied flycatchers, significant mismatches between food supply and demand have been found recently [46].

Another important finding in our study is the link between the departure decision at the non-breeding sites and the matching of the spring green-up at the respective breeding sites about 3800 km away. Individuals, who left their non-breeding sites early, matched spring green-up at the breeding site more accurately. Even if departure from Africa is mainly driven by photoperiod, local conditions potentially fine-tune the decision, but we failed to verify this link. However, this would enhance the relevance of phenological trends at the non-breeding sites and their potential implications for demographic rates (survival, reproductive success) at later times at sites far away.

There are several methodological issues that potentially have confounded our findings – the accuracy of light-based positioning and the relation between NDVI data and higher-level productivity that we have implicitly assumed: Light-based positions naturally have a relatively large inaccuracy, e.g. compared to GPS positions, especially in woodland species [27]. Therefore, the sites identified often comprise large areas, possibly including unsuitable habitats. Although we have explicitly excluded unsuitable habitats (desert, bare or sparsely vegetated ground) and thus, reduced this source of error considerably, our method is necessarily relatively coarse and might still confound finer-scale patterns. Many studies investigating the reliance of migrants on environmental conditions used NDVI data as a potential cue for timing (e.g. [22]) or as a proxy for food abundance in non-herbivorous species, i.e. species foraging on higher trophic levels (e.g. [53]). Although NDVI and primary production have been related explicitly [28,54], its relation to productivity at higher trophic levels requires further specification (e.g. [55]). By using temperature data stemming from an atmospheric model for modelling insect phenology and by pooling the thermal requirements of several insect taxa, we cannot predict small-scale variations in resource phenology. However, the approach allows for modelling the general insect abundance as for food for adults and offspring in insectivorous birds across large areas where field data are often impossible to achieve.

## Conclusions

Our findings suggest that nightingales used cues other than local phenology for their departure decisions from non-breeding grounds.

The influence of global warming on vegetation phenology is well-studied in temperate and polar regions [56,57]. Much less is known about phenological trends and their potential role in decision making in birds for tropical or subtropical regions such as Sahelian Africa [58]. This, however, is urgently required if we aim at further increasing our understanding of the causal relations between the environmental conditions, a decision the migrant takes at local scale and their consequences during several periods of the annual cycle in migrants. Such mechanistic links between vastly distant places are key for assessing the demographic consequences of habitat and climatic changes [59] and thus, for efficient management and conservation of migratory species.

## Competing interests

The authors declare that they have no competing interests.

## Authors’ contributions

SH and SB were involved in the design of the study. TE performed the data analysis. All authors have written and approved the manuscript.

## Supplementary Material

Additional file 1 Tables with general information about the study populations, the individual migration schedules as well as the parameters used for data analysis and calibration. Click here for file

Additional file 2 Description of the light-level geolocator data analysis procedure. Click here for file
