# Supplementary material for: Individual migration timing of common nightingales is tuned with vegetation and prey phenology at breeding sites
Source: BMC Ecol. 2014 Mar 21;14:9. doi: 10.1186/1472-6785-14-9 (PMC3999983; doi:10.1186/1472-6785-14-9)
Supplement: Additional file 1 — Tables with general information about the study populations, the individual migration schedules as well as the parameters used for data analysis and calibration. [file 1472-6785-14-9-S1.docx]

Additional file 1

Table A1: The four study populations in Bulgaria, Italy and France where nightingales were captured and equipped with geolocators in 2009, and recaptured in 2010. Lon.: longitude; Lat.: latitude; # e: number of birds equipped; # r: number of birds recaptured; # a: number of birds analysed (complete recording of spring migration until recapture).

| Study site | Population | Lon. | Lat. | # e | # r | # a |
| --- | --- | --- | --- | --- | --- | --- |
| Kavarna | Bulgaria | 28.33° E | 43.42° N | 35 | 1 | 0 |
| Brodilivo | Bulgaria | 27.85° E | 42.08° N | 65 | 11 | 4 |
| Campotto | Italy | 11.83° E | 44.58° N | 100 | 6 | 1 |
| Petite Camargue Alcasienne | France | 07.53° E | 47.62° N | 100 | 11 | 4 |

Table A2: Compilation of individual migration schedules and corresponding measures for food and vegetation phenology. Departure from non-breeding site (T_DEP_NB_), arrival at stopover site (T_ARR_S_), departure from stopover site (T_DEP_S_) and arrival at breeding site (T_ARR_B_) are given as day of the year. Stopover (T_DEP_S_ - T_ARR_S_) and total migration duration (T_ARR_B_ - T_DEP_NB_), the time intervals between arrival and spring green up at the breeding site (T_ARR_B_ - t_SPRING_), between arrival and the date when insects start to develop at the breeding site (T_ARR_B_ - t_BDT_) as well as between the end of the offspring’s peak food requirement and the end of larvae availability (T_ARR_B_* - t_DD_) are given in days.

| Total | | Non-breeding | Stopover | | | Breeding sites | | | |
| --- | --- | --- | --- | --- | --- | --- | --- | --- | --- |
| Bird | T_ARR_B_ - T_DEP_NB_ | T_DEP_NB_ | T_ARR_S_ | T_DEP_S_ | T_DEP_S_ - T_ARR_S_ | T_ARR_B_ | T_ARR_B_ - t_SPRING_ | T_ARR_B_ - t_BDT_ | T_ARR_B_* - t_DD_ |
| B1 | 25 | 86 | 90 | 101 | 11 | 111 | 16 | 10 | 5 |
| B2 | 27 | 81 | 91 | 101 | 10 | 108 | 13 | 7 | 8 |
| B3 | 31 | 85 | 89 | 108 | 19 | 116 | 21 | 15 | 0 |
| B4 | 20 | 84 | 87 | 98 | 11 | 104 | 9 | 3 | 12 |
| I1 | 23 | 84 | 91 | 103 | 12 | 107 | 18 | 1 | 26 |
| F1 | 33 | 84 | 87 | 107 | 20 | 117 | 14 | 10 | 24 |
| F2 | 32 | 78 | 91 | 106 | 15 | 110 | 7 | 3 | 31 |
| F3 | 27 | 77 | 90 | 104 | 14 | 104 | 1 | -3 | 37 |
| F4 | 22 | 83 | 98 | 103 | 5 | 105 | 2 | -2 | 36 |
| ø | 26.9 | 82 | 90 | 103 | 13.0 | 109 | 11.3 | 4.9 | 19.9 |

Table A3: The sun elevation angles (SEA, in degrees below the horizon) resulting from the in-habitat calibration to the coordinates of the respective breeding site, the distance for filtering (in kilometers) as well as the days (in days) and quantile (as percentage) parameters for the determination of the stationary periods.

| Bird ID | SEA | distance | days | quantile |
| --- | --- | --- | --- | --- |
| B1 | -4.5 | 1000 | 3 | 90 |
| B2 | -4.8 | 1000 | 3 | 90 |
| B3 | -4.1 | 1000 | 3 | 90 |
| B4 | -5.1 | 1000 | 3 | 90 |
| I1 | -5.5 | 1000 | 3 | 90 |
| F1 | -3.2 | 1000 | 3 | 90 |
| F2 | -5.8 | 1000 | 3 | 90 |
| F3 | -6.5 | 1000 | 3 | 90 |
| F4 | -6.0 | 1000 | 3 | 90 |
| ø | -5.1 | 1000 | 3 | 90 |
