# Supplementary material for: Individual migration timing of common nightingales is tuned with vegetation and prey phenology at breeding sites
Source: BMC Ecol. 2014 Mar 21;14:9. doi: 10.1186/1472-6785-14-9 (PMC3999983; doi:10.1186/1472-6785-14-9)
Supplement: Additional file 2 — Description of the light-level geolocator data analysis procedure. [file 1472-6785-14-9-S2.docx]

Additional file 2

## We used the light-level threshold method for positioning (Hill 1984), using software GeoLocator (Swiss Ornithological Institute). Additionally, we used the R-package GeoLight as described in Lisovski & Hahn 2012:

## I. Determination of sun events

We determined sun set and sun rise from daily light measurements of 5 min intervals using the software GeoLocator (see Figure 1). Baseline light intensity was zero for these SOI-GDL 1.0, thus we used light level threshold of three (on a unit less arbitrary light intensity scale). Sun rise sun set times can also be determined in GeoLight using the function twilightCalc.


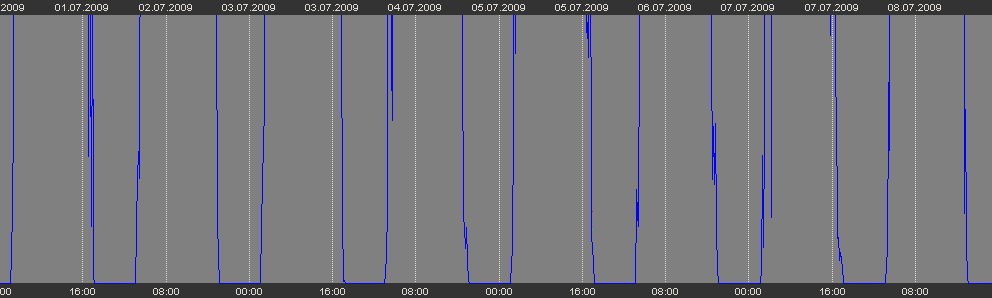

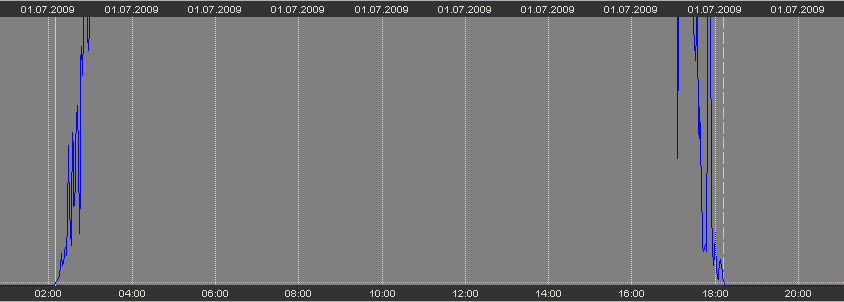


Figure 1: Illustration of the determination of sun events in screenshots of the GeoLocator software. For each day we defined sun rise (solid yellow line) as the time point when the light intensity (solid blue line) exceeds the light level threshold (yellow dotted line) in the morning and sun set (dashed yellow line) as the time point when the light intensity falls below the light level threshold in the evening.

## II. Identification of stationary periods

We identified stationary periods using similarities in subsequent sun events along the time axis with the changeLight function of GeoLight. In this process, the probability of change for every time point in the sunrise and sunset data series (Figure 2, centre panel) is calculated separately (Figure 2, bottom panel). To account for individual differences in behaviour the 90% quantile of change point probabilities is used as a threshold. This means that only those 10% of the sun event data with the highest change point probabilities were considered as change points where conditions and/or behaviour changes (for sun rise and sun set separately). The change points separate stationary periods from periods of movement (Figure 2, top panel). We set the minimal length of stationary periods to 3 days.


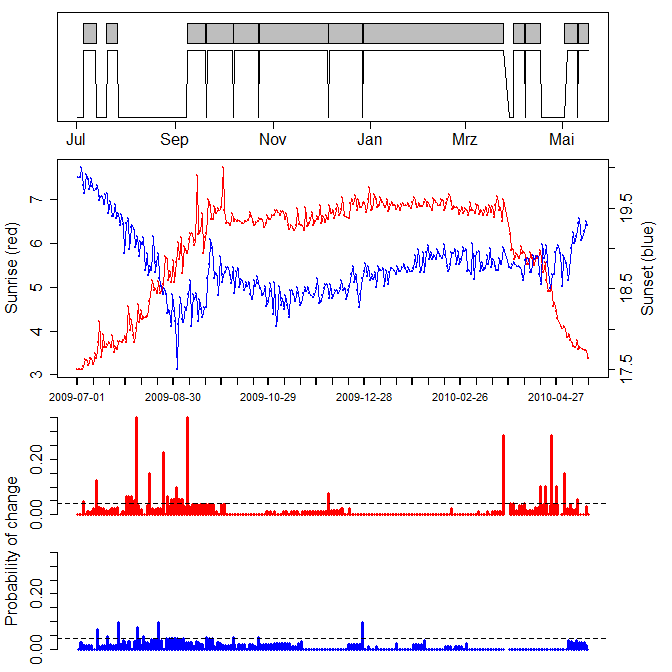


Figure 2: The four panels of the output graph of the changeLight function visualize the procedure of the discrimination of stationary periods. For every time point in the sunrise (red) and sunset (blue) event (centre panel) the probability of change is calculated (bottom panels). Probability thresholds (black dashed lines) are set at the 90% quantile, resulting in only 10% of highest probabilities of change surmount the threshold. Change points with probabilities above the threshold affect the temporal migration pattern (top panel), i.e. they define the time when tagged individuals shifted between stationary periods (grey bars) and periods of movement (gaps).

## III. Calibration

We started with a dual calibration approach for each individual - a Hill-Ekstrom calibration for non-breeding periods, i.e. minimisation of variance in latitude and an in-habitat calibration approach for the known breeding sites (Lisovski et al. 2012). The core requirement for Hill-Ekstrom calibration, i.e. invariable shading intensity within a focal period was not fulfilled. Thus, in-habitat calibration results were used for non-breeding and stopover sites.
By in-habitat calibration we can correct for local shading conditions and individual habitat clutter in the positioning process by assuming same conditions during migrations as during calibration. With the function getElevation we calculate individual sun elevation angles (see Figure 3) during the time of residence on its breeding site (after arrival, timing is determined by changeLight, see above). Assuming that conditions and behaviour during spring migration match conditions and behaviour in the breeding habitat in spring, we applied these individual sun elevation angles for the whole spring journey.


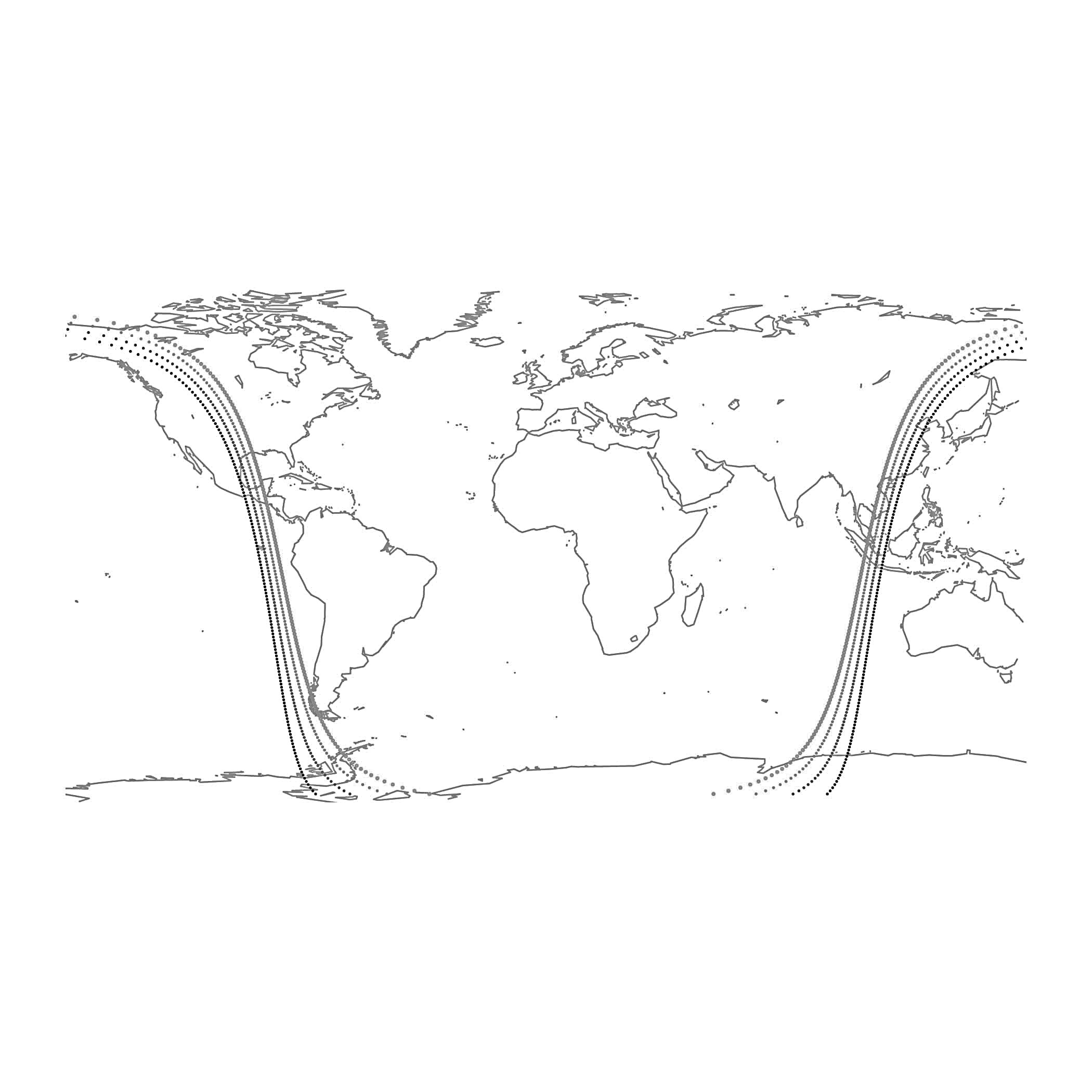


Figure 3: Schematic graph showing the effect of changing sun elevation angle on the latitudinal positioning (solid horizontal lines) of a particular day length. Dotted lines indicate sun rise (left) and sun set (right) for four different sun elevation angles.

## IV. Positioning

Geographical coordinates were calculated with the function coord, which applies astronomical equations to convert two succeeding sun events to latitude and longitude using the sun elevation angle determined in the calibration process (see above). Because position cannot be calculated during periods of almost equal day length across earth (equinox periods; the line of sun events are vertical in contrast to Figure 3) the function output can contain NA values in latitude, but not in longitude. Changes in the behaviour during sunrise/set as well as irregular shading can result in unrealistic positions (see Lisovski et al. 2012)
